# Micro- and nanoplastics and fecal microbiota transplantation: a conceptual framework linking gut dysbiosis and intestinal barrier dysfunction

**DOI:** 10.1016/j.ijpx.2026.100699

**Published:** 2026-11-03

**Authors:** Qiuyue Tu, Huizhen Li, Jiaxin Li, Zelin Feng, Xinyue Wei, Hetong Zhang, Qinghua Yi, Haojia Lin, Yanru Zhang, Yueting Li, Xiaoqin Li, Yu Wang, Ye Chen, Qi Su, Lei Wang, Xiaocang Cao

**Affiliations:** a Department of Gastroenterology and Hepatology, Tianjin Medical University General Hospital, Tianjin Medical University, Tianjin, China; b Department of Gastroenterology, Shenzhen Hospital, Southern Medical University, Shenzhen, Guangdong, China; c Shenzhen Clinical Research Center for Digestive Disease, Shenzhen, Guangdong, China; d Microbiota I-Center, Hong Kong SAR, China; e Department of Medicine and Therapeutics, Chinese University of Hong Kong, Hong Kong SAR, China; f MOE Key Laboratory of Pollution Processes and Environmental Criteria, College of Environmental Science and Engineering, Nankai University, Tianjin, China; g School of Public Health, Tianjin Medical University, Tianjin, China

**Keywords:** Fecal microbiota transplantation, Microplastics, Nanoplastics, Iatrogenic exposure, Gut microbiota, Intestinal barrier

## Abstract

Fecal microbiota transplantation (FMT) is increasingly recognized as a promising therapeutic option for microbiota-related disorders. Its efficacy may be affected by factors related to both donor microbiota and the recipient’s intestinal barrier. Micro- and nanoplastics (MNPs), emerging environmental contaminants, have been recognized for their detrimental impacts on intestinal health. Existing studies have shown that MNPs can induce gut dysbiosis, impair intestinal barrier, and alter mucosal immune responses. These findings raise the possibility that MNPs may influence biological processes relevant to FMT, including donor microbiota characteristics and the recipient intestinal microenvironment. However, evidence of MNPs-induced intestinal toxicity does not establish impaired donor microbial engraftment or reduced therapeutic FMT efficacy, and the proposed relationship remains untested. Furthermore, although MNPs release from medical materials has been reported, whether FMT procedures contribute to measurable MNPs exposure remains unknown. This review summarizes current evidence on MNPs-associated intestinal health and proposes a potential mechanistic framework linking MNPs exposure to FMT outcomes, through alterations in gut microbiota, and intestinal barrier. Future studies are needed to validate this proposed framework by directly investigating the effects of MNPs exposure on donor microbial engraftment, microbial functional recovery, and clinical outcomes following FMT.

## Graphical abstract

**Figure:**
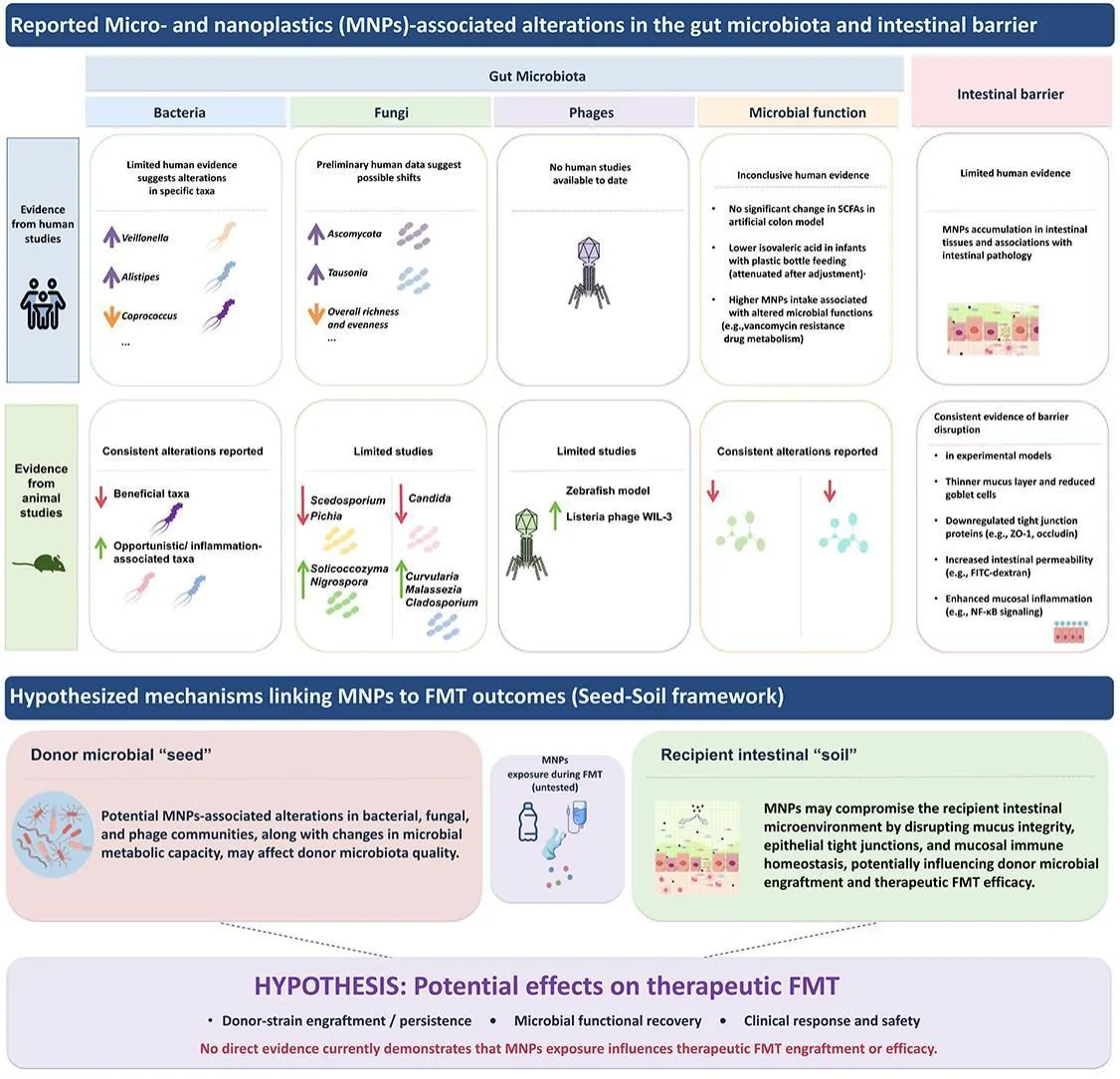

## 1 Introduction

The human gut microbiome constitutes a complex ecosystem influenced by both host and environmental factors. Gut dysbiosis is increasingly regarded as a context-dependent alteration in the composition and/or functional capacity of the gut microbial community that is associated with a particular host condition or disease (Coutry et al., 2024; Hooks and O'Malley, 2017; Winter and Bäumler, 2023). Gut dysbiosis has been implicated in a wide range of diseases (Shen et al., 2025). By transferring microbiota from the stool of eligible donors into the intestines of individuals with diseases, fecal microbiota transplantation (FMT) facilitates the re-establishment of a balanced gut microbiome, ameliorates disorders associated with gut dysbiosis. In recent years, FMT has emerged as a promising gut dysbiosis-based therapeutic strategy, with growing evidence supporting its potential applications in gastrointestinal and extraintestinal diseases (Hou et al., 2025; Wang et al., 2022b).

FMT therapeutic efficacy is shaped by factors from both the donor and the recipient (Porcari et al., 2023). The donor microbiota is acknowledged as critical to FMT success. Emerging evidence indicates that recipient-related factors are also crucial. In particular, the condition of the recipient’s intestinal barrier during transplantation impacts the engraftment and functionality of the donor microbiota, thereby influencing treatment outcomes. The intestinal barrier consists of four interconnected layers (Neurath et al., 2025): (I) the microbial barrier; (II) the chemical barrier, primarily composed of mucus; (III) the physical barrier, including epithelial cells and tight junction proteins; and (IV) the immunological barrier, including gut-associated lymphoid tissue and antimicrobial peptides. Successful colonization of the donor microbiota (the “seed”) depends on the recipient’s intestinal barrier (the “soil”), which includes factors such as inflammatory status and barrier integrity (Cebi and Yilmaz, 2025; Zhang et al., 2023a). Gut dysbiosis is often accompanied by impairment of the intestinal barrier, a condition highly prevalent in patients undergoing FMT, such as individuals with inflammatory bowel disease (IBD), and metabolic syndrome (Cebi and Yilmaz, 2025; Qian et al., 2025). The exacerbation of the patient’s intestinal barrier dysfunction during the peri-transplantation period may not only impair microbial donor microbiota engraftment but also exacerbate inflammation, thereby diminishing the therapeutic efficacy of FMT.

Emerging evidence, derived predominantly from animal studies and supported by a limited number of human observational studies, suggests that micro- and nanoplastics (MNPs), may perturb both sides of this equation by altering microbiota composition and compromising intestinal barrier. These findings raise a biologically plausible question about whether MNPs exposure could influence factors relevant to FMT. MNPs, defined as plastic particles smaller than 5 mm, including microplastics (MPs, size: 1 μm–5 mm) and nanoplastics (NPs, size: <1 μm), are increasingly recognized as pervasive environmental pollutants with potential impacts on gut health (Vethaak and Legler, 2021). Clinical observations have suggested that MNPs may be a risk factor for diseases such as cardiovascular disease and IBD (Marfella et al., 2024; Yan et al., 2022). The gastrointestinal tract is a primary target of MNPs-induced toxicity, where they may directly interact with gut microbiota and the intestinal barrier (Cao et al., 2024; Pan et al., 2024; Schwarzfischer and Rogler, 2022).

Critically, FMT has appeared in this literature in two distinct roles that must not be conflated. The gut microbiota is a demonstrated mediator of MNPs-induced intestinal and extra-intestinal toxicity: in several studies, FMT was used purely as an experimental tool to establish this causality, by transferring microbiota from MNPs-exposed donor animals into previously healthy, MNPs-naive recipients, which then developed adverse intestinal and distal-organ phenotypes (Wen et al., 2022; Zhang et al., 2024a; Zhang et al., 2023b). These findings support a potential causal contribution of MNPs-associated microbial alterations to the observed adverse effects. However, such experiments differ fundamentally from therapeutic FMT, which aims to modify the recipient’s intestinal microbial ecosystem to achieve clinical benefit (Kazemian et al., 2020). Evidence of microbiota-mediated toxicity should not be interpreted as evidence that MNPs exposure impairs donor microbial engraftment or reduces therapeutic FMT efficacy. Rather, MNPs-associated alterations in microbial communities and intestinal barrier provide a mechanistic rationale for investigating this possibility, which remains to be directly tested.

Despite growing evidence of MNPs-associated intestinal toxicity, several critical knowledge gaps remain regarding its potential relevance to therapeutic FMT. First, direct evidence linking MNPs exposure to therapeutic FMT outcomes is lacking, as no published studies, to our knowledge, have specifically evaluated this relationship. Second, although MNPs-induced alterations in gut microbiota, intestinal barrier integrity, and inflammatory responses have been documented, how these biological changes might affect donor microbiota characteristics, recipient intestinal receptivity, and subsequent microbial engraftment remains unclear. Third, the clinical relevance of MNPs exposure as a potential modifier of FMT outcomes remains unknown, particularly given the lack of prospective human studies integrating quantitative exposure assessment with longitudinal microbiological and clinical outcomes. By integrating evidence of MNPs-associated gut microbiota alterations, intestinal barrier dysfunction, and inflammatory and metabolic disturbances, Shruti et al. (Shruti et al., 2025) explored the potential mechanistic links between MNPs exposure and diabetes. Building on these insights, the present review extends the discussion beyond established MNPs-associated biological effects by raising a previously underexplored question: whether MNPs exposure may influence the efficacy of therapeutic FMT. To explore this possibility, we introduce a “seed-soil” framework that draws on existing evidence of MNPs-associated alterations in gut microbiota and intestinal barrier function to hypothesize how such effects, if occurring in FMT donors and recipients, might influence donor microbiota characteristics, recipient intestinal receptivity, microbial engraftment, and therapeutic outcomes.

Accordingly, this narrative review aims to propose biologically plausible mechanisms linking MNPs exposure to potential changes in FMT outcomes, draw attention to this underexplored research question, and encourage further experimental and clinical investigations to evaluate these hypotheses.

## 2 Methods

A non-systematic literature review was conducted using peer-reviewed articles written in English and published before July 2026, retrieved from the PubMed and Web of Science databases. Given that the efficacy of FMT is closely associated with gut health, and that research on the interplay between therapeutic FMT and MNPs is lacking, the literature search focused on studies investigating the effects of MNPs on intestinal health. Our search strategy included terms: “microplastics”, “nanoplastics”, “human health”, “intestinal”, “gut ”, “gut microbiota”, “gut barrier”, “intestinal barrier”, and “fecal microbiota transplantation”. We also used a “snowballing” approach in which additional studies were selected from references of articles identified by the above searches.

To further characterize the available human evidence on MNPs exposure and gut microbiota, we conducted a focused evidence mapping of original human studies by searching PubMed and Web of Science in July 2026 using two search strategies targeting publications: “(microplastic OR nanoplastic OR "micro- and nanoplastic") AND (gut microbiota OR gut microbiome OR intestinal microbiota OR intestinal microbiome OR fecal microbiota OR faecal microbiota OR microbiome) AND (human OR patient OR volunteer)”, and “(microplastic OR nanoplastic) AND (gut microbiota OR gut microbiome OR microbiome) AND (cohort OR observational OR cross-sectional OR clinical)”. Studies were considered eligible for the human evidence mapping if they: (1) reported original research involving human participants or human-derived biological samples; (2) investigated MNPs exposure, occurrence, or burden in relation to human gut microbiota; and (3) provided data on intestinal microbial composition, diversity, or functional characteristics. Studies were excluded if they were reviews, or commentaries, or meta-analyses, exclusively animal or in vitro investigations without human-derived microbiota data, environmental microbiology studies unrelated to the human gut, studies without relevant gut microbiota outcomes, or publications that did not provide eligible original data. The searches identified 1,085 records, of which 365 duplicates were removed, leaving 720 records for screening. Subsequently, 316 reviews, meta-analyses, or commentaries, 280 animal or in vitro studies, 11 unrelated publications, five human studies without gut microbiota data, 88 environmental microbiology studies, and one study with insufficient data for extraction were excluded. A total of 19 human studies were retained for the evidence mapping and summarized in Table 1.

**Table 1:** The human gut microbiota dysbiosis associated with micro and nano-plastics.

Reference	Feces Source	Major results about gut microbiota
(Gao et al., 2025)	healthy adults (n=39)	No significant differences in alpha and beta diversity were found between the low- and high- MPs^1^ exposure groups. The relative abundance of *Faecalibacterium prausnitzii* was significantly higher in the low MPs exposure group compared with the high MPs exposure group. MPs correlated positively with unclassified species belonging to *Enterobacteriaceae* and *Escherichia coli*, as well as a negative correlation with *Faecalibacterium prausnitzii.* MPs were positively correlated with genes encoding invasion-related virulence factors. A positive correlation was found between microplastics and microbial transporter systems. A positive correlation was found between microplastics and genes encoding microplastic biodegradation enzymes.
(Hong et al., 2024)	high MPs exposure college students (HME, n=53); low MPs exposure college students (LME, n=68)	The difference in the richness and diversity of bacterial communities were not significant. The HME group had increased levels of *Collinsella*, *Erysipelotrichaceae, Clostridium, Desulfovibrionaceae* and *Blautia_hydrogenotrophica*, while there was a decrease in *Allisonella.* The richness, evenness and composition of the fungal species were not significant. Many pathogenic fungi such as *Ascomycota, Tausonia* and *Dothideomycetes* increased in the HME group.
(Tilves et al., 2023)	infants (n=70)	Infants who were plastic bottle fed less than every feeding versus every feeding had lower fecal microbiota alpha diversity at 3 months and lower isovaleric acid concentration at 3 months, but these results were attenuated following adjustment for infant diet. Plastic bottle frequency at 3 months was associated with all beta diversity metrics at 3 months, however, these results were not significant after adjustment for key covariates and infant diet. There were no significant associations between plastic bottle frequency at 3 months and microbiome alpha and beta diversity at 12 months In the 3-month microbiota, infants who were plastic bottles less than every feeding versus every feeding had significantly lower *Collinsella aerofaciens*. In the 12-month microbiota, infants who were plastic bottles less than every feeding versus every feeding had significantly lower *Parabacteroides merdae* and *Faecalibacterium (ASV110)*.
(Schwenger et al., 2024)	healthy liver donors (n=6); obese subjects with a normal liver (n=6); obese subjects with metabolic dysfunction-associated steatohepatitis (n=11)	MPs fibers correlated positively with *Bifidobacterium* and negatively with *Lachnospiraceae*.
(Zhang et al., 2022)	high MPs exposure healthy adults (HME, n=20); low exposure healthy adults (LME, n=20)	Shannon indices showed no significant difference between HME and LME groups. At the phylum level, the HME group had significantly lower *Proteobacteria* and *Campilobacterota* than the LME group. At the genus level, the HME group had significantly lower *Bifidobacterium*, *Streptococcus*, and *Sphingomonas* than the LME group, while had significantly higher *Fusobacterium*, *Coprococcus*, *[Ruminococcus]_torques_group*, *Dorea*, and *Butyricicoccus* than the LME group.
(Zha et al., 2023)	occasionally consuming TFDPC^2^ college students (AP, n=170); frequently consuming TFDPC college students (BP, n=162); never consumed TFDPC college students (NC, n=58)	The gut microbiota richness was determined at similar levels in AP, BP and NC cohorts. The diversity and evenness were both greater in the AP and BP cohorts than in the NC cohort. A difference was shown in the gut microbiota composition of the three cohorts. Compared with the NC cohort, multiple gut bacteria were associated with the AP and BP cohorts, among which *Faecalibacterium, Collinsella* and *Dorea* were the three intestinal bacteria most associated with the AP cohort, while intestinal *Collinsella, Dorea* and *Agathobacte*r were most associated with BP cohort.
(Zhang et al., 2024b)	DPT^3^ exposure postgraduate students (n=30); DPT non-exposure postgraduate students (n=30); DPT post-exposure postgraduate students (n=27)	Alpha diversity and Beta diversity were not significant between the DPT group and the non-DPT group. The post-exposure group showed significantly lower diversity compared with the DPT and non-DPT groups. At the phylum level, compared with the non-DPT group, the DPT and post-exposure groups had a lower abundance of *Bacteroidota* and a higher abundance of *Actinobacteriota*. At the genus level, compared with the non-DPT group, DPT and post-exposure groups had a lower abundance of *Faecalibacterium* and a higher abundance of *Blautia*.
(Ke et al., 2023)	high microplastics exposure preschool children (HME, n=28); low microplastics exposure preschool children (LME, n=27)	The HME group had higher Chao1 and Observed_species indices than the LME group. Shannon and Simpson indices showed no significant difference between HME and LME groups. The HME group had lower *Lactobacillales, Rikenellaceae, Alistipes, Streptococcus,* and *Streptococcaceae* than the LME group.
(Liu et al., 2022)	18 mother−infant pairs	Polystyrene was inversely related to the Chao index of meconium microbiota. MPs were positively correlated with *Streptococcus, Clostridia_UCG-014, Treponema*, and *Prevotellaceae_NK3B31_group*, while inversely correlated with *Pelomonas*.
(Ma et al., 2025)	45 older adults: L-group (≤ 19.36 particles/g, n=15); medium-exposure (19.36–56.34 particles/g, n=15), and high-exposure (≥ 56.34 particles/g, n=15) groups	MPs altered β-diversity without affecting α-diversity, increased MDI and decreased GMHI in a dose-dependent manner. Reduced *Firmicutes* and *Blautia*, and increased *Escherichia-Shigella* and *Bifidobacterium* in the H-group; compared with the L-group, the M-group showed lower *Veillonell*a and higher *Erysipelotrichaceae_UCG-003* and *Alistipes*, whereas the high-exposure group showed higher *Klebsiella* and *Enterobacter*; compared with the M-group, the H-group exhibited higher *Escherichia-Shigella* and *Klebsiella* but lower *Anaerostipe*s and *Erysipelotrichaceae_UCG-003*. MPs induced differential metabolites enriched in ABC transporters, amino acid, vitamin, glutathione, and protein metabolism pathways, which were significantly correlated with gut microbiota alterations.
(Jiang et al., 2024)	healthy hematopoietic stem cell transplantation donors (n = 14)	Higher fecal MPs exposure in HSCT donors was associated with reduced abundances of *Rikenellaceae* and *Oscillospiraceae*. Higher MPs exposure was associated with decreased fecal hypoxanthine levels in donors. Fecal MPs levels in donors were negatively correlated with recipient survival after hematopoietic stem cell transplantation.
(Kao et al., 2026)	68 children under the age of 6 years	No significant changes in α-diversity or overall microbial community structure. Higher MPs exposure was associated with enrichment of *Bacilli*, whereas *Bacteroides*, *Flavonifractor*, and *Alistipes* were enriched in the low MPs group. MPs exposure was positively associated with *Streptococcus salivarius* abundance.
(Song et al., 2026)	high MPs exposure college students (HG, n=33); low exposure college students (LG, n=33)	MPs was associated with altered gut microbial diversity and community structure, with increased species diversity and significant separation in β-diversity. At phylum-level, HG was characterized by enrichment of *Gemmatimonadota*. At Genus-level, HG was characterized by increased *Evtepia*. Species-level shifts included enrichment of potentially harmful taxa in HG, including *Dialister invisus* and Evtepia *gabavorous.*
(Feng et al., 2025a)	high MPs exposure college students (H, n = 12); low MPs exposure college students (L, n = 12)	No significant differences in α- or β-diversity. *Shewanellaceae*, *Shewanella*, and *Megasphaera* were enriched in the H group, whereas *Peptostreptococcaceae* and *Romboutsia* were enriched in the L group. Nanoplastics-positive samples exhibited a greater number of OTUs. Nanoplastics-negative samples were enriched in *Proteobacteria* and *Desulfobacterota*, with increased *Desulfovibrio*, *[Eubacterium]_eligens_group*, and *Negativibacillus*, and decreased *Butyricicoccus*.
(Liu et al., 2026)	preschool children (n=80)	High MPs exposure was associated with altered gut microbiota composition, with increased microbial diversity, distinct β-diversity, lower Firmicutes/Bacteroidetes ratio, and enrichment of *Bacteroidaceae*, *Streptococcaceae*, *Prevotellaceae*, *Oscillospiraceae*, and *Muribaculaceae*. Functional alterations involved metabolic pathways related to macronutrients, vitamins, and bioactive compounds.
(Zhao et al., 2026)	high MPs exposure healthy adults (H-MPs, n=76); low exposure healthy adults (L-MPs, n=75)	H-MPs exposure was associated with increased α-diversity, while β-diversity remained unchanged. *Ruminococcus* and *Escherichia* showed lower relative abundances in the H-MPs group, although these differences were not significant after false discovery rate correction. A total of 507 microbial species differed between groups before false discovery rate correction. Pathways related to chemical carcinogenesis, neuromuscular disorders, and neurodegenerative diseases were enriched in the H-MPs group.
(Nugrahapraja et al., 2022)	22 healthy adults	No significant association between microplastic exposure and overall gut microbiota composition, diversity, richness, or gut health-related genes. However, specific plastic types were associated with selected bacterial taxa: high-density polyethylene was negatively correlated with *Bacteroides*; polypropylene was positively correlated with *Roseburia* and *Clostridium* but negatively with *Prevotella copri*; polystyrene showed the opposite associations. Plastic-degrading enzyme genes were also detected, suggesting potential microbial adaptation to microplastic exposure.
(Thanga Velu et al., 2026)	healthy controls (n=35); patients with colonic polyps (n=35)	Higher polyurethane and polyvinyl chloride levels were detected in polyp tissues and stool, respectively. Polyp patients exhibited reduced gut microbial richness and evenness. Mucin-degrading bacteria (*Parabacteroides distasonis, Phocaeicola vulgatus, Bacteroides thetaiotaomicron*) and the mucin-producing bacterium *Blautia* were enriched in polyps, suggesting microbial remodeling of the mucosal barrier. *Fusobacterium* with pathogenic potential was frequently detected in polyp tissues.
(Peng et al., 2026)	31 patients with colorectal cancer	Peri-tumor polyamide 66 was positively correlated with *Fusobacterium simiae* and *Tepidibacter formicigenes*. Tumor polyamide 66 was associated with the sulfate-reducing anaerobe *Lagierella massiliensis*. Blood polystyrene was positively associated with *Cronobacter sakazakii* and *Sphingomonas panacis*. Peri-tumor polystyrene was associated with increased abundance of *Eubacterium sp. CAG:202*, *Clostridium sp. CAG:122*, and *Blautia sp. MSK20.85*. Tumor polystyrene was positively associated with *Neisseria elongata*, *Fusobacterium sp. CM22*, and *Myoviridae sp. CtTfA5*. Tumor *polyvinyl chloride* was associated with enrichment of *Streptococcus spp.* and *Tissierella spp.* Microplastic exposure was associated with functional remodeling of the tumor-associated microbiome, with enrichment of cancer-, immune/inflammation-, and metabolism-related microbial pathways.

1 MPs: microplastics.

2 TFDPC: take-away food in disposable plastic containers.

3 DPT: Disposable plastic tableware.

## 3 MNPs exposure

### 3.1 Daily exposure

Plastics are unavoidable around the world. MNPs exposure can be broadly categorized into daily and iatrogenic sources. In daily exposure, MNPs can enter the human body through oral ingestion, inhalation, and dermal contact (Luo et al., 2025). Among these routes, the gastrointestinal tract serves as the primary route for MNPs, mainly through contaminated food and drinking water, as well as the ingestion of dust (Chakraborty et al., 2025; Heo et al., 2025; Wen et al., 2024b). A study conducted in Mumbai, India, estimated that the average daily intake of MNPs per person was approximately 2,012 ± 598 particles, derived from drinking water (382 ± 205 particles), air (594 ± 269 particles), and food (1,036 ± 493 particles) (Yadav et al., 2022). Estimated MNPs from hot and cold beverages in the UK was 1.7 MPs/kg/day for females and 1.6 MPs/kg/day for males, exceeding the estimated intake from drinking water alone (1 MP/kg/day) (Al-Mansoori et al., 2025). In addition to direct contamination of food through contact with plastic packaging, environmental MNPs can also enter the human food chain indirectly. For example, plants can absorb atmospheric MNPs through their leaves (Li et al., 2025c). Coastal fish can bioaccumulate marine MNPs through the food chain (Liu et al., 2025). Fortunately, most ingested particles are excreted in feces. In healthy mice, 99% of orally administered fluorescent polystyrene (PS) NPs excreted within 24 hours (Tian et al., 2025b). However, a fraction of ingested MNPs can evade gastrointestinal clearance and undergo intestinal translocation. Smaller-sized MNPs, particularly those below approximately 150 μm, exhibit a greater potential for epithelial uptake through size-dependent mechanisms, including transcellular transport, M-cell-mediated transcytosis, and, under conditions of intestinal barrier disruption, paracellular passage through compromised tight junctions (Dávalos et al., 2026; Li et al., 2024).

### 3.2 Potential iatrogenic exposure

MNPs have been detected in healthcare environments, and medical devices and consumables have been identified as potential sources of particle release. Increased airborne MNPs have been detected in operating rooms during the working day (Field et al., 2022). Several studies have reported MNPs release from plastic-containing medical devices and consumables, including masks, infusion sets, syringes, plastic drug containers, and wound dressings, under specific experimental or usage conditions (Cui et al., 2024; Jin, 2022; Kim et al., 2025; Li et al., 2025a; Liang et al., 2022; Tuna and Taş, 2025). Zheng et al. (Zheng et al., 2024) showed that polyvinyl chloride (PVC) infusion tubing releases PVC MNPs directly into fluids during intravenous administration. These findings indicate that healthcare environments and certain medical devices may contribute to potential MNPs exposure. However, environmental detection and experimentally demonstrated particle release do not necessarily indicate substantial patient exposure, and the actual contribution of these sources to the overall MNPs burden remains uncertain.

Given that FMT involves the collection, processing, storage, and administration of donor-derived fecal material, similar medical-related sources of MNPs may theoretically be relevant to FMT procedures. Several potential exposure pathways can be hypothesized. First, plastic consumables used during donor stool collection, processing, and storage may introduce particles into fecal preparations if shedding occurs. Second, gastrointestinal delivery devices, including nasoenteric and rectal tubes and endoscopic instruments, may potentially release particles during FMT administration. Although Wu et al. (Wu et al., 2025) reported findings suggesting a possible association between repeated gastrointestinal endoscopy and intestinal MNPs accumulation, these observations do not establish particle shedding from medical instruments during FMT. Third, MNPs potentially present in donor stool may persist through processing and subsequently be transferred to recipients. However, these possibilities are extrapolated from general medical-device studies rather than direct evidence from FMT procedures. Whether MNPs are released from FMT-related devices, introduced during fecal preparation, or retained in final transplant preparations remains unknown.

Furthermore, the underlying gastrointestinal conditions of some FMT recipients may theoretically influence the fate of MNPs entering the intestinal tract. Patients with IBD, or metabolic syndrome, exhibit varying degrees of intestinal barrier damage (Mansouri et al., 2025; Mishra et al., 2025). Emerging evidence suggests that individuals with disrupted intestinal barriers may retain or absorb more MNPs than healthy counterparts. Yan et al. (Yan et al., 2022) suggested that in patients with IBD, a condition characterized by intestinal barrier disruption, the fecal MNPs was significantly higher than in healthy controls (41.8 vs. 28 particles/g), suggesting altered clearance or increased retention under inflammatory conditions. Experimental studies have shown prolonged gastrointestinal retention of PS NPs in constipated and colitis mouse models (Tian et al., 2025b), while intestinal barrier injury may facilitate particle translocation under certain experimental conditions (Liang et al., 2021). Together, these findings raise the possibility that pre-existing gastrointestinal disorders may modify the retention and translocation of ingested MNPs in some FMT recipients, although direct evidence in this population remains lacking.

Collectively, existing evidence of MNPs contamination in healthcare environments and particle release from medical devices provides a rationale for hypothesizing that FMT recipients may also experience medical-related MNPs exposure during treatment. However, this possibility remains unverified in FMT settings. Further experimental investigations and clinical studies involving FMT participants are needed to identify potential exposure sources, quantify MNPs in donor stool and final transplant preparations, and determine whether FMT procedures contribute to additional MNPs exposure.

## 4 Potential mechanistic links between MNPs exposure and FMT outcomes

### 4.1 Seed-soil model in FMT

In FMT, the “seed-soil” model provides a useful theoretical framework for understanding therapeutic mechanisms (Fig. 1). In this model, the donor microbiota acts as the “seed”, while the recipient’s intestinal barrier serves as the “soil”. From the perspective of the seed, the diversity of donor microbiota, the ecological adaptability at the strain level, and the abundance of key functional taxa (e.g., short-chain fatty acid-producing bacteria, bile acid-metabolizing bacteria, anti-inflammatory symbionts) are closely associated with the clinical efficacy of FMT (Kazemian et al., 2020; Wilson et al., 2021).

**Fig. 1:**
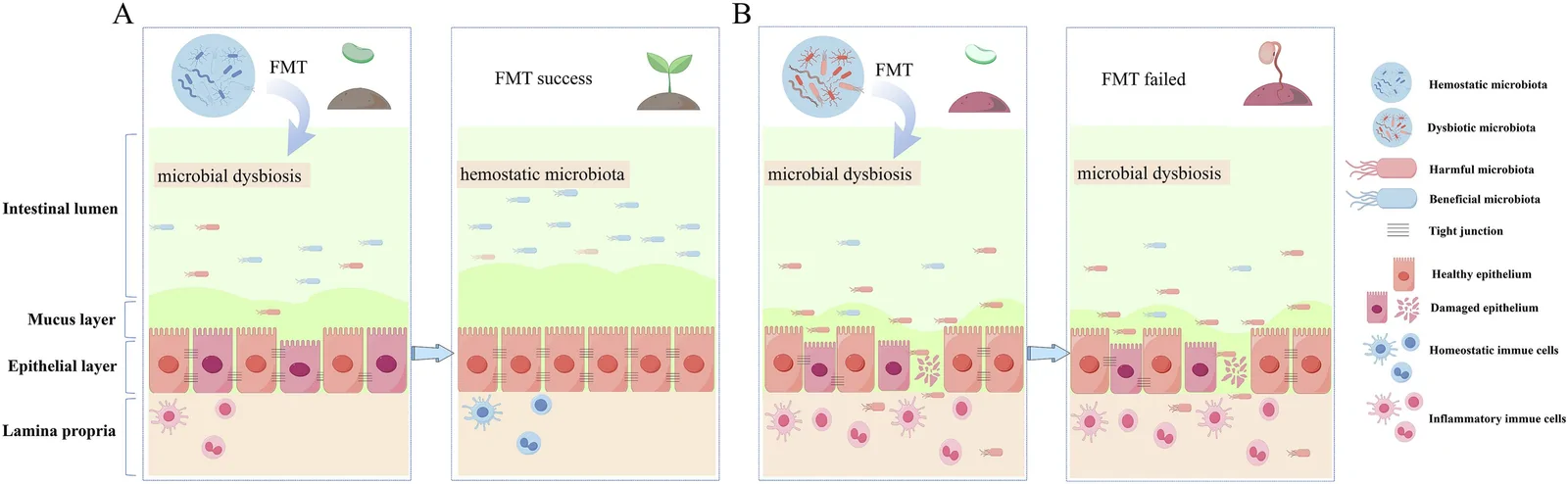
The “seed–soil” model of fecal microbiota transplantation (FMT). (A) Successful FMT: a diverse and functional donor microbiota (“seed”) engrafts in a permissive recipient environment (“soil”), characterized by a relatively preserved intestinal barrier function, including mucus layer integrity, epithelial structure, and controlled immune responses, which enabling microbial colonization and promoting restoration of microbiota homeostasis. (B) Failed FMT: impaired donor microbiota “seed” and/or a non-permissive intestinal “soil”, characterized by mucus depletion, epithelial damage, disrupted tight junctions, and excessive inflammatory responses, limit microbial engraftment and perpetuate dysbiosis.

Additionally, the patient’s intestinal barrier integrity and stability, plays a critical role in the success of microbial engraftment. Studies have shown that recipients with impaired intestinal barriers, such as reduced mucus thickness, increased permeability, or dysregulated immunity, may have limited capacity to support donor microbiota engraftment (Danne et al., 2021; Porcari et al., 2023). Zhang et al. (Zhang et al., 2023a) reviewed gut damage mechanisms in non-alcoholic fatty liver disease (NAFLD) and the prospects of microbiota-based interventions, noting that restoring the mucus layer may enhance probiotic colonization and therapeutic efficacy. Before FMT, patients with lower intestinal inflammation tend to achieve more successful donor engraftment and symptom relief (Danne et al., 2021). However, FMT success is a multidimensional outcome, encompassing donor-strain engraftment, microbial functional recovery, and clinical response, which are related but distinct and may not necessarily occur concurrently. Nevertheless, the “seed-soil” model provides a simplified conceptual framework for exploring how MNPs exposure may influence FMT outcomes. Based on this “seed–soil” framework, we hypothesize that MNPs-induced alterations in gut microbiota and intestinal barrier may have potential implications for the donors’ “microbial seed” and recipients’ “intestinal soil” involved in FMT.

### 4.2 MNPs-associated alterations in gut microbiota

A healthy donor gut microbiota introduces beneficial microbes whose proliferation and metabolic activity suppress pathogens, which is crucial for FMT success (Hou et al., 2025). Current evidence indicates that MNPs alter gut microbial composition and function (Fig. 2). However, evidence from human studies remains limited. Table 1 summarizes the 19 human studies identified through our focused evidence mapping that investigated associations between MNPs exposure and the human gut microbiota. The available evidence is predominantly observational, with limitations including relatively small sample sizes, heterogeneous study populations, differences in exposure assessment and microbiome profiling methods, and potential confounding factors. Moreover, the reported microbial alterations are inconsistent across studies. For example, Nugrahapraja et al. (Nugrahapraja et al., 2022) and Gao et al. (Gao et al., 2025) found no significant differences in overall microbial diversity or composition, despite reporting associations with selected bacterial taxa. Beyond taxonomic changes, several studies have reported associations between MNPs exposure and microbial metabolic characteristics, including short-chain fatty acids, amino acid-related metabolites, functional pathways, and antibiotic resistance genes (Tilves et al., 2023; Ma et al., 2025; Gao et al., 2025; Liu et al., 2026). These findings suggest that MNPs-associated microbial alterations may involve functional characteristics relevant to intestinal homeostasis. From the perspective of FMT, microbial community composition and metabolic functions are potentially relevant to donor microbiota characteristics, microbial engraftment, and the restoration of intestinal homeostasis. Therefore, the reported associations provide a biological rationale for investigating whether MNPs exposure might influence FMT-related processes. However, these observational findings cannot establish causal relationships between MNPs exposure and gut microbial alterations, and changes in microbial composition or functional potential do not necessarily indicate impaired microbial function. Importantly, none of the 19 human studies directly evaluated the relationship between MNPs exposure and donor microbiota engraftment, recipient microbial recovery, or clinical outcomes following FMT. Thus, the potential implications of MNPs-associated microbial alterations for therapeutic FMT remain hypothetical and require direct experimental and clinical investigation.

**Fig. 2:**
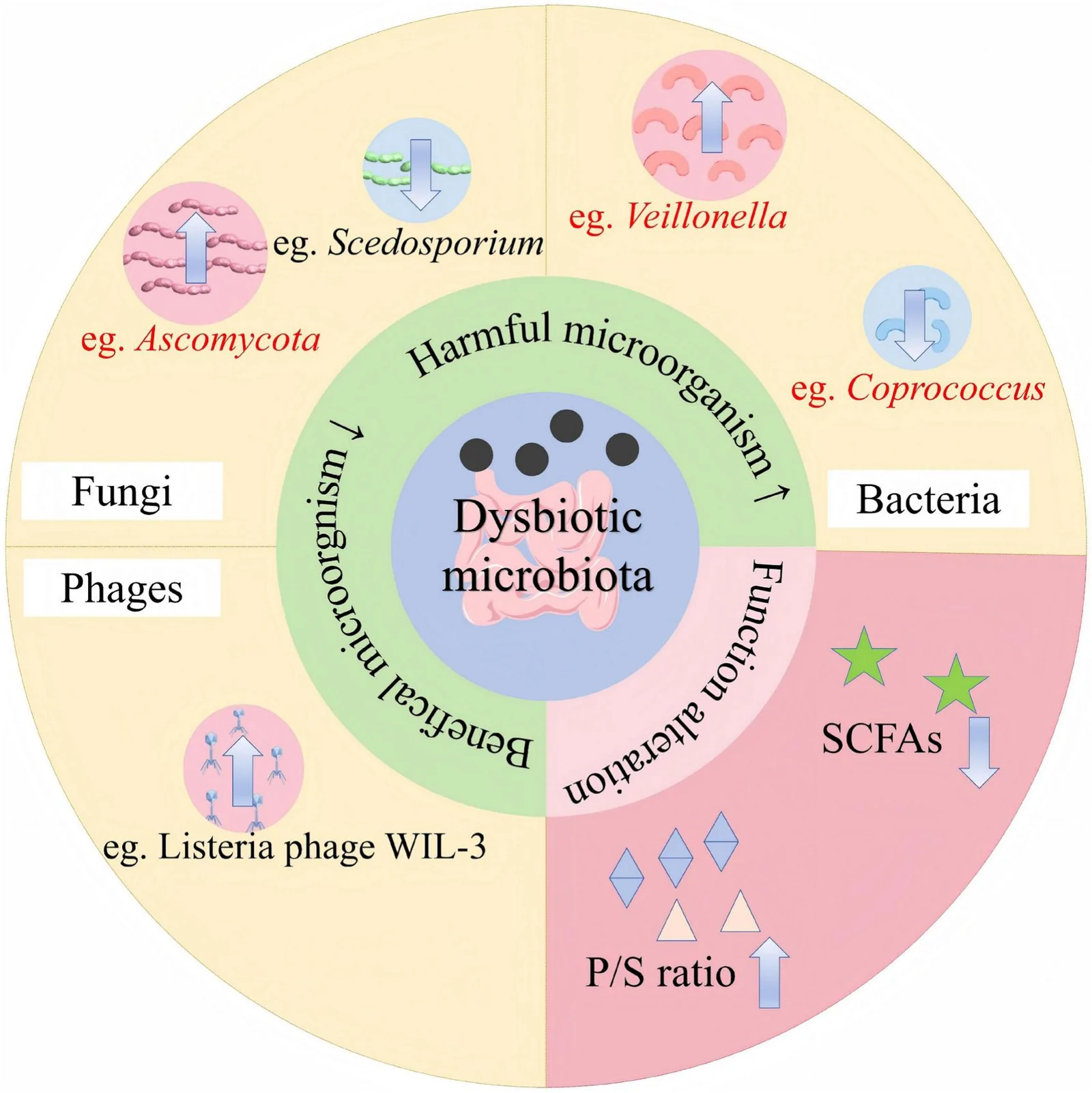
Micro- and nanoplastics (MNPs) induce gut microbiota dysbiosis by altering microbial structure, including the bacteriome, mycobiome, and virome. Concurrently, MNPs-induced dysbiosis is associated with functional alterations, including reduced short-chain fatty acid (SCFA) production and an increased primary-to-secondary bile acids (P/S) ratio. (Red text indicates evidence from human studies, whereas black text indicates evidence from animal studies.) (For interpretation of the references to color in this figure legend, the reader is referred to the web version of this article.)

#### 4.2.1 Changes in gut microbial composition

Gut microorganisms can be broadly classified according to their functional relationships with the host. Beneficial commensals contribute to intestinal homeostasis through metabolic activities, colonization resistance, and immune regulation, whereas pathogens have the potential to cause host damage or disease. Opportunistic pathogens, including certain resident pathobionts, may cause disease under permissive conditions, such as impaired host defenses or disruption of the intestinal microbial ecosystem (Dey and Ray Chaudhuri, 2023; Tewari and Dey, 2024). However, these categories are not absolute, as microbial functions depend on host physiological conditions, microbial interactions, and species- or strain-specific characteristics (Yu et al., 2026). This context-dependent behavior also applies to intestinal fungi, which may contribute to immune homeostasis while exhibiting pathogenic potential under certain conditions (Hill and Round, 2024; Ost and Round, 2023). Accordingly, MNPs-associated changes in microbial abundance should not be interpreted as direct evidence of altered microbial function or impaired FMT efficacy without functional validation. To provide a clearer overview of these context-dependent microbial functions, Table 2 summarizes the functional classifications, potential roles in intestinal homeostasis and dysbiosis, and reported MNPs-associated alterations of representative gut bacteria and fungi.

**Table 2:** Functional classification, potential intestinal roles, and micro- and nanoplastics (MNPs)-associated alterations of representative gut bacteria and fungi.

Reference	Taxon	Context-dependent functional potential	Possible intestinal role	MNPs-associated observation
	BACTERIA			
Zhang et al., 2024c	*Allobaculum*	Reported homeostatic / metabolic potential / SCFA-associated	SCFA-associated genus; possible homeostasis support	↓; PS-MNPs exposure decreased *Allobaculum* abundance in mice, which was associated with alterations in lipid- and amino acid-related metabolites
Hong et al., 2024	*Coprococcus*	Reported homeostatic / metabolic potential / butyrate-associated	Butyrate-related metabolic functions;	↓; Negatively associated with fecal MPs counts in young adults
Hong et al., 2024	*Veillonella*	Context-dependent; Reported disease-associated potential	Lactate metabolism; associations vary by species and host	↑; Positively associated with fecal MPs counts in young adults
Hong et al., 2024	*Alistipes*	Context-dependent	Species-dependent associations with host metabolism and inflammation	↑; Positively associated with fecal MPs counts in young adults
Zhang et al., 2022	*Ruminococcus torques group*	Context-dependent commensal / Reported disease-associated potential	Potential mucin use; disease/FMT association is independent evidence	↑; Higher relative abundance in the intestinal microbiota of individuals with high MPs exposure than in those with low exposure.
Zhang et al., 2022	*Fusobacterium*	Context-dependent commensal / Reported disease-associated potential	Some species linked to mucosal inflammation; not all genus members pathogenic	↑; Higher relative abundance in the intestinal microbiota of individuals with high MPs exposure than in those with low exposure.

	FUNGI			
Gao et al., 2023	*Scedosporium*	Opportunistic potential (species-dependent)	Certain species can cause opportunistic infections, particularly in immunocompromised individuals. Its physiological role in the gut remains unclear.	↓: Following exposure to 0.05-0.1 μm PS particles in mice
Gao et al., 2023	*Pichia*	Context-dependent / Insufficiently resolved	Some species participate in lipid metabolism and may contribute to microbial interactions; their effects on intestinal homeostasis remain insufficiently characterized.	↓; Following exposure to 0.05-0.1 μm PS particles in mice
Gao et al., 2023	*Solicoccozyma*	Uncertain functional significance	Primarily an environmental yeast genus; increased abundance has been associated with gastric cancer, although its intestinal function and pathogenicity remain unclear.	↑; Following exposure to 0.05-0.1 μm PS particles in mice
Gao et al., 2023	*Nigrospora*	Reported disease-associated fungus; pathogenic role unconfirmed	Higher abundance has been positively associated with clinical disease activity indices in patients with ulcerative colitis, without establishing a causal role.	↑; Following exposure to 0.05-0.1 μm PS particles in mice
Gao et al., 2023	*Candida*	Commensal / Reported disease-associated potential	Certain species are common fungal colonizers of mucosal surfaces and may become pathogenic under conditions of impaired host immunity or microbial imbalance.	↓; Following exposure to 9-10 μm PS in mice
Gao et al., 2023	*Curvularia*	Opportunistic potential / unclear gut role	Certain species can cause opportunistic infections; increased intestinal abundance has been reported in patients with non-alcoholic fatty liver disease, although its gut-specific pathogenic role remains uncertain.	↑; Following exposure to 9-10 μm PS in mice
Gao et al., 2023	*Malassezia*	Context-dependent commensal / Reported disease-associated potential	Certain species, particularly M. restricta, have been implicated in intestinal inflammatory responses and Crohn’s disease-associated dysbiosis.	↑; Following exposure to 9-10 μm PS in mice
Gao et al., 2023	*Cladosporium*	Environmental-associated fungus / Opportunistic potential	Commonly found in environmental sources; some species are opportunistic pathogens, but their contribution to intestinal homeostasis or dysbiosis remains poorly defined.	↑; Following exposure to 9-10 μm PS in mice
Hong et al., 2024	*Tausonia*	Uncertain functional significance	Human gut role and pathogenic status uncertain	↑; Higher relative abundance in the high-MPs-exposure group of young adults
Hong et al., 2024	*Ascomycota*	NOT classifiable as a single functional group	Broad phylum includes diverse commensals and pathogens	↑; Higher relative abundance in the high-MPs-exposure group of young adults

Important caveat: Assignments below describe reported potential functions and are not fixed taxonomic traits. Functional phenotype is strain-specific, host-state dependent, and cannot be inferred solely from genus-level relative abundance shifts observed in MNPs exposure studies. No entry should be interpreted as a stable “beneficial” or “pathogenic” label.

Bacteria Increasing evidence indicates that MNPs reshape gut bacterial communities, although current evidence is derived predominantly from animal studies. MNPs exposure alters gut bacterial diversity and community composition, although the affected taxa vary considerably among studies depending on MNPs characteristics and disease context (Jiménez-Arroyo et al., 2023; Xie et al., 2022). Broad phylum-level metrics such as the F/B ratio should be interpreted with caution. Similarly, changes in individual bacterial taxa should not be assumed to indicate beneficial or detrimental effects without corresponding functional evidence. Several animal studies have reported reductions in beneficial bacteria and enrichment of opportunistic or inflammation-associated bacteria following MNPs exposure (Chen et al., 2025b; Lo et al., 2026; Yin et al., 2025). For example, *Allobaculum*, a short-chain fatty acids (SCFAs)-producing genus associated with intestinal homeostasis, was markedly reduced following PS MNPs exposure in mice (Pujo et al., 2021; Zhang et al., 2024c). Human evidence remains scarce but suggests similar trends, with higher fecal MNPs burdens associated with increased *Veillonella* and *Alistipes* and decreased *Coprococcus* (Hong et al., 2024). Separately, associations between these taxa and metabolic disorders or FMT responses have been reported in other clinical contexts. For instance, the *Veillonella* and *Alistipes* have been linked to obesity (Hong et al., 2024), and *Coprococcus* is a key butyrate producer and is considered as a beneficial bacterium associated with improved outcomes of FMT in treating type 2 diabetes (Ng et al., 2022). However, these observations originate from different populations and study designs, and findings from human observational studies may be influenced by potential confounding factors. Therefore, they do not establish that MNPs-associated alterations in these taxa predict donor microbiota engraftment or FMT efficacy. Furthermore, the functional significance of individual taxa may vary across species, strains, and ecological contexts.

Fungi Emerging evidence suggests that MNPs exposure may alter gut fungal community composition, although current findings are largely derived from animal studies. In mice, Gao et al. (Gao et al., 2023) reported particle size-related differences in gut fungal responses to PS particles, with PS NPs affecting 41 fungal genera compared with 33 genera following PS MPs exposure. Specifically, PS NPs exposure was associated with decreased relative abundances of *Scedosporium* and *Pichia* and increased abundances of *Solicoccozyma* and *Nigrospora*, whereas PS MPs exposure was associated with reduced *Candida* abundance and increased *Curvularia*, *Malassezia*, and *Cladosporium* abundances. Although some of these genera have been associated with intestinal inflammation or metabolic disorders in independent clinical studies (Borgo et al., 2017; Lam et al., 2019; Qiu et al., 2017; You et al., 2021), these associations do not establish that MNPs-induced fungal alterations contribute to disease development or progression.

Human evidence remains limited. Hong et al. (Hong et al., 2024) observed a nonsignificant trend toward reduced gut fungal richness and evenness among individuals with higher MNPs levels, alongside differences in the relative abundances of certain fungal taxa, including *Ascomycota* and *Tausonia*. However, the functional implications of these observations remain unclear, particularly given the differences in exposure assessment, host characteristics, and taxonomic resolution across studies. Overall, current evidence suggests that MNPs exposure may be associated with changes in gut fungal community composition, but whether these alterations affect intestinal homeostasis, donor microbiota characteristics, or therapeutic FMT outcomes remains unknown.

Phages Although research on MNPs-induced phages alterations is still in its infancy, limited animal studies have begun to explore this area. For instance, in a zebrafish model, 21-day exposure to 15 μg/L PS NPs led to a significant increase in Listeria phage WIL-3 (KT319643), whose host *Listeria monocytogenes* is a known human pathogen (Klumpp and Loessner, 2013; Teng et al., 2023). However, this effect was not observed at a higher concentration (150 μg/L), suggesting a non-linear, concentration-dependent response (Teng et al., 2023). Furthermore, the findings are specific to the experimental model and exposure conditions. Whether MNPs-associated virome alterations occur in humans or influence donor microbiota engraftment and clinical outcomes following FMT remains unknown.

#### 4.2.2 Changes in gut microbial metabolism

The SCFAs and secondary bile acids (SBAs) are especially relevant to FMT, as they are vital for restoring gut microbiome and aiding recipient recovery post-FMT (Danne et al., 2021; Kazemian et al., 2020). However, accumulating evidence from animal studies indicates that MNPs lead to a reduction in the production of SCFAs and SBAs of gut (Qiao et al., 2021; Tu et al., 2023). Alterations in microbial metabolic capacity represent a biologically plausible pathway through which MNPs exposure could affect the functional characteristics of the donor’s gut microbiota. However, evidence that MNPs-induced metabolic changes directly impair FMT outcomes is currently lacking.

Experimental studies in murine models revealed significant downregulation of SCFAs following PS MNPs’ exposure, with more pronounced effects observed at smaller particle sizes and higher concentrations (Kang et al., 2025; Zhang et al., 2023c). MNPs-associated disturbances in bile acid metabolism have also been observed in animal models. For example, Wen et al. (Wen et al., 2024a) reported reduced fecal SBA levels and an increased primary-to-secondary bile acid ratio following PS MPs exposure, accompanied by alterations in hepatic bile acid-related gene expression and gut microbial composition. Similarly, Chen et al. (Chen et al., 2025a) reported that long-term exposure to PS MPs was associated with disrupted bile acid metabolism, accumulation of conjugated bile acids in the colon, epithelial apoptosis, and intestinal inflammation. These findings support the involvement of microbial and host metabolic disturbances in MNPs-associated intestinal toxicity, although they do not establish that the observed metabolic changes would compromise the engraftment or therapeutic activity of transplanted microbiota. The potential relevance of these metabolic changes to FMT is supported by separate studies investigating microbiota-mediated therapeutic mechanisms. For example, microbial bile acid metabolism has been implicated in the therapeutic effects of FMT for recurrent Clostridioides difficile infection, and reduced bile acid metabolic capacity has been associated with poorer treatment responses in relevant clinical settings (Collins et al., 2022; Kazemian et al., 2020).

Evidence on the effects of MNPs on human gut microbiota function remains limited and inconclusive. Exposure to polyethylene (PE) MPs in the Mucosal Artificial Colon model revealed no statistically significant changes in SCFAs production (Fournier et al., 2023). Furthermore, in an observational infant study, Tilves et al. (Tilves et al., 2023) observed that infants fed with plastic bottles less frequently had lower concentrations of isovaleric acid (a type of SCFA) at 3 months compared to those fed with plastic bottles at every feeding, but these results were attenuated following adjustment for infant diet. It is regrettable that this research did not calculate the exposure dose of MNPs in infants, and overlooked the impact of MNPs from other exposure routes. It highlights the need for more rigorous human studies to elucidate the complex relationship between MNPs and SCFAs. Another human study revealed that higher MNPs intake significantly altered gut microbiota function, enhancing vancomycin resistance, selenocompound metabolism, and drug-metabolizing enzyme activity (Hong et al., 2024).

In summary, experimental evidence indicates that MNPs exposure can alter gut microbial composition and metabolic characteristics, while human studies have identified heterogeneous associations that require further validation. Importantly, microbiota-transfer experiments have demonstrated that microbial communities from MNPs-exposed animals can transmit certain adverse phenotypes to recipient animals, supporting a causal contribution of the altered microbiota to specific MNPs-associated toxic effects (Jiang et al., 2024; Wu et al., 2026; Zhai et al., 2026). However, these experiments were designed to investigate the microbiota-mediated toxicity of MNPs, rather than the therapeutic efficacy of FMT. The transmission of adverse phenotypes does not establish impaired donor-strain engraftment, reduced microbial ecosystem restoration, or treatment failure following therapeutic FMT.

Based on these findings, we hypothesize that MNPs exposure may influence FMT outcomes by modifying the donor-derived “microbial seed”. Specifically, donors with higher MNPs exposure may harbor distinct microbial communities compared with donors with lower exposure, which could potentially alter microbial engraftment, ecological reconstruction, and therapeutic efficacy after FMT. However, direct evidence regarding the effects of MNPs exposure on the gut microbiota of FMT donors remains lacking.

### 4.3 MNPs-associated alterations in the intestinal barrier

Successful FMT relies not only on a healthy “microbial seed” from the donor but also on gut barrier in the recipient, which serves as a selective interface regulating microbial engraftment, immune homeostasis, and inflammation (Porcari et al., 2023). Disruption of this barrier may hinder the establishment and stability of transplanted microbiota. Increasing evidence from animal studies indicates that MNPs compromise the intestinal barrier, including microbial, chemical, physical, and immunological defenses (Fig. 3). These findings raise the possibility that MNPs exposure could modify the recipient intestinal environment in ways relevant to FMT. However, evidence of MNPs-induced barrier alterations should be distinguished from evidence of impaired therapeutic engraftment or FMT efficacy, which has not been directly established. In the following sections, we summarize current evidence regarding the impact of MNPs on different aspects of the intestinal barrier and discuss their potential relevance to FMT.

**Fig. 3:**
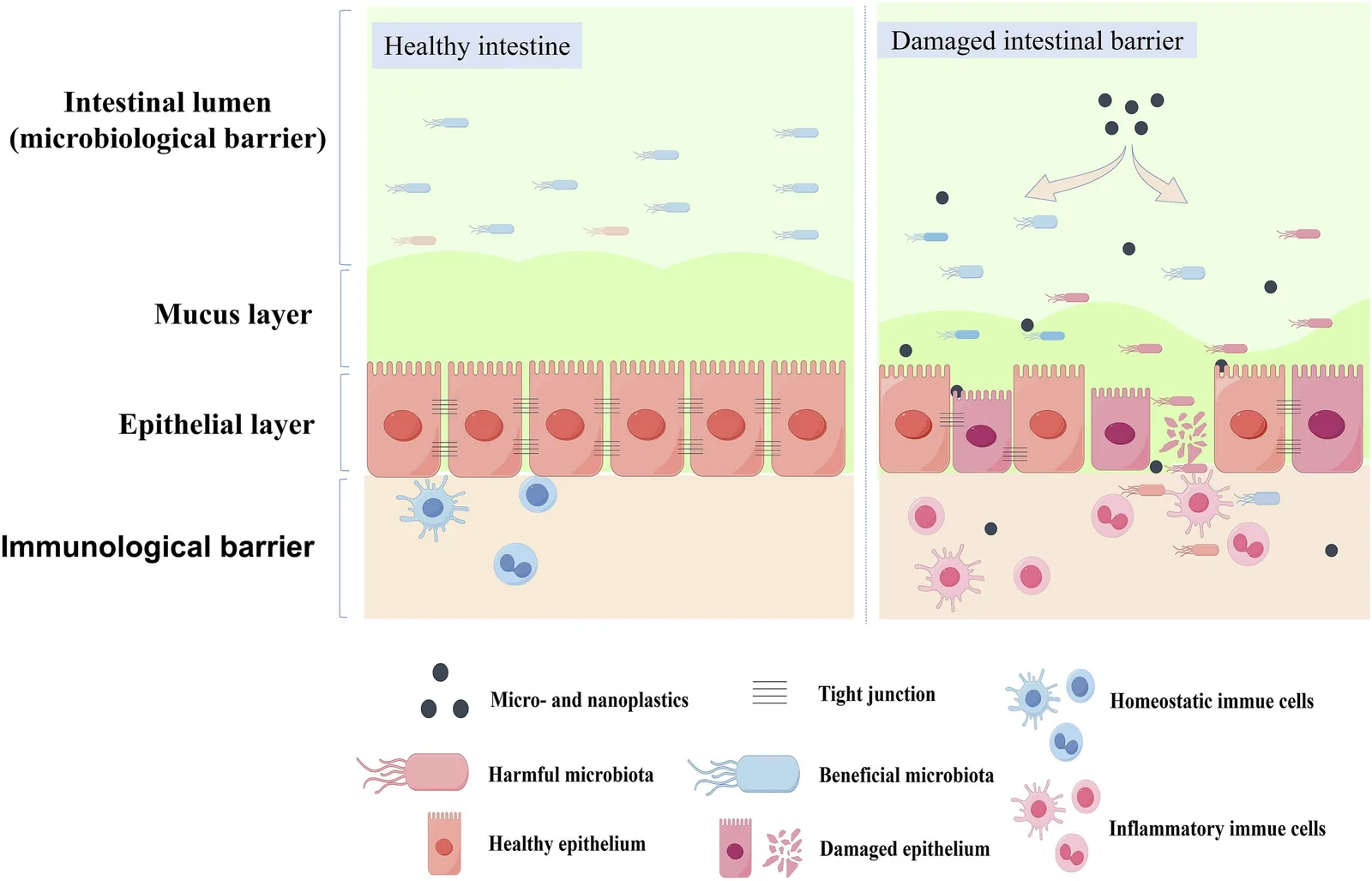
Micro- and nanoplastics (MNPs) impair intestinal barrier integrity across microbial, chemical, physical, and immunological layers.

#### 4.3.1 Potential effects of MNPs on the microbial barrier

Existing studies have demonstrated that MNPs exposure alters gut microbiota, accompanied by increased disease severity. Based on these observations, we hypothesize that that MNPs may not only influence the donor’s “microbial seed” but also aggravate existing microbial barrier disruption in patients receiving FMT, potentially affecting the engraftment of donor-derived microbiota following FMT. Given that recipient microbiota characteristics have been associated with FMT outcomes across different diseases (Danne et al., 2021; Porcari et al., 2023), we consider metabolic syndrome and IBD as representative examples to explore this possibility.

In metabolic syndrome, patients with failed FMT treatment typically exhibit higher microbiota diversity, characterized by elevated levels of *Ruminococcus torques* compared to responders before FMT (Kootte et al., 2017). In a separate experimental setting, exposure to 10 mg/L PS MPs worsened metabolic disorders induced by a high-fat diet in mice, with 16S rRNA sequencing revealing an increased α-diversity of the gut microbiota (Zhai et al., 2024). Additionally, human studies have shown that individuals with higher MNPs’ exposure exhibit a greater abundance of the *Ruminococcus torques group* in their feces (Zhang et al., 2022). Similarly, in IBD, the enrichment of *Fusobacterium* before FMT in patients with UC and the colonization of *Enterobacteriaceae* in patients with CD have been associated with unfavorable FMT outcomes (Porcari et al., 2023). Separately, evidence suggests that MNPs exposure increases the relative abundance of potentially pathogenic bacteria, including *Fusobacterium* in human populations (Zhang et al., 2022).

Studies demonstrate that recipient baseline microbiota characteristics can be associated with FMT outcomes and, independently, that MNPs exposure can be associated with alterations in microbial composition in disease models. Although these findings suggest some overlap in the microbial characteristics reported across FMT and MNPs exposure studies, they originate from different populations, experimental conditions, and disease contexts. Therefore, these cross-study observations do not establish that MNPs-associated microbial alterations predict donor microbial engraftment or therapeutic FMT outcomes. Whether MNPs exposure modifies the recipient microbial environment in ways that influence FMT responses remains to be determined.

#### 4.3.2 Potential effects of MNPs on the chemical and physical barriers

Evidence from animal studies indicated that MNPs disrupts mucus integrity and epithelial barrier function. Meng et al. (Meng et al., 2025) found that PS MPs reduced mucus viscosity, leading to thinning and loss of the mucus layer. Further studies showed that MNPs was associated with reduced mucus secretion and decreased expression of mucin genes (Muc1, Muc2, Muc3) in the colon (Ji et al., 2023). Beyond changes in mucus quantity, MNPs may also modify its protective properties. In reconstituted mucin hydrogels, Henkel et al. (Henkel et al., 2026) demonstrated that PS NPs interacted with mucin networks and altered their selective permeability. These findings suggest that MNPs may compromise the chemical barrier not only by changing mucus quantity but also by modifying its material properties and interactions with substances passing through it.

At the epithelial level, MNPs-associated barrier dysfunction has been characterized by altered tight-junction protein expression, increased permeability, and histopathological injury. For example, Zeng et al. (Zeng et al., 2024) demonstrated that PS MPs increased intestinal permeability and reduced the expression of ZO-1, occludin, and claudin-1 in mice. Similarly, Li et al. (Li et al., 2026) reported that PS fibers and beads decreased the expression of epithelial junctional proteins and MUC2, with more pronounced colonic injury following fiber exposure. These findings establish structural and functional manifestations of MNPs-associated barrier damage.

MNPs-induced epithelial injury involves several interconnected cellular processes, including membrane perturbation, oxidative stress, mitochondrial dysfunction, metabolic dysregulation, DNA damage, and regulated cell death (Das, 2023; Møller and Roursgaard, 2023; Wu et al., 2024; Wu et al., 2020). These mechanisms may compromise epithelial integrity by disrupting cellular homeostasis, reducing epithelial renewal, and promoting cell loss. Nevertheless, their relative contributions vary according to particle size, polymer composition, surface characteristics, exposure conditions, and biological models.

The plasma membrane represents an initial interface between MNPs and intestinal epithelial cells. Positively charged MNPs interact with negatively charged phospholipids in cell membranes, increasing membrane permeability, altering membrane potential, and even forming pores (Dai et al., 2022; Wang et al., 2022a). Using supported lipid bilayers and giant unilamellar vesicles together with cell-based validation, Wang et al. (Wang et al., 2026) demonstrated that PS NPs reduced membrane lipid mobility and promoted cholesterol-dependent endocytosis, thereby facilitating the uptake of otherwise poorly permeable substances. These findings indicate that MNPs can modify membrane biophysical properties and cellular transport processes. However, particle internalization is not necessarily required for epithelial injury. Tejeda et al. (Tejeda et al., 2026) observed altered mitochondrial morphology and impaired membrane integrity in Caco-2 cells exposed to gastric-acid-treated polyethylene particles, despite the absence of detectable cellular internalization. Together, these observations suggest that both membrane-associated interactions and intracellular particle effects may contribute to epithelial dysfunction.

Following particle-cell interactions, oxidative stress may represent an important link between initial membrane perturbation and downstream epithelial dysfunction. Excessive reactive oxygen species (ROS) can damage cellular lipids, proteins, and organelles, including mitochondria, thereby disrupting energy metabolism and cellular homeostasis. In a human intestinal epithelial coculture model, Zhang et al. (Zhang et al., 2026b) demonstrated that gastrointestinally digested PET MPs caused mitochondrial damage, ATP depletion, and sustained activation of AMP-activated protein kinase (AMPK). These alterations were accompanied by reduced epithelial proliferation, DNA double-strand breaks, and impaired barrier integrity. A complementary study by Zhang et al. (Zhang et al., 2026a) identified impaired glucose metabolism and abnormal mitochondrial Complex I hyperactivation following exposure to digested PET MPs, resulting in excessive ROS generation and disrupted ATP homeostasis. These findings suggest that MNPs-induced mitochondrial dysfunction may involve maladaptive metabolic reprogramming rather than a uniform suppression of mitochondrial activity. Collectively, mitochondrial injury and metabolic disturbances may weaken epithelial maintenance by limiting energy availability and activating stress-responsive pathways.

In addition to mitochondrial dysfunction, MNPs exposure may disturb endoplasmic reticulum (ER) homeostasis and cellular repair processes. Ardicli et al. (Ardicli et al., 2026) reported that PET MNPs induced oxidative and ER stress, reduced epithelial viability, and decreased transepithelial electrical resistance in an intestinal gut-on-a-chip model. These observations suggest that organellar stress may accompany the deterioration of epithelial barrier function. DNA damage and cell-cycle disturbances represent additional mechanisms through which MNPs may affect epithelial renewal. Zhang et al. (Zhang et al., 2026b) linked sustained AMPK activation to p53/p21-associated cell-cycle regulation and increased DNA damage. Tian et al. (Tian et al., 2025a) further demonstrated that PS NPs activated PI3K/AKT/mTOR signaling in AOM/DSS-treated mice and Caco-2 cells, accompanied by disrupted lipid metabolism, oxidative stress, and DNA damage, thereby promoting colitis-associated cancer progression. Xie et al. (Xie et al., 2023) demonstrated that PS MPs overactivated Notch signaling, promoting colonic epithelial overproliferation and impairing goblet cell differentiation. Similarly, Shaoyong et al. (Shaoyong et al., 2023) reported that benzo[a]pyrene-loaded aged PS MPs exacerbated colonic barrier injury through oxidative stress-associated Notch activation. However, oxidative stress does not invariably produce detectable genotoxicity. Huovinen et al. (Huovinen et al., 2026) reported increased ROS production and reduced cellular viability following nylon MPs exposure without significant genotoxic effects under the experimental conditions. Therefore, oxidative stress, DNA damage, and impaired proliferation should be considered related but distinct cellular outcomes.

Persistent cellular stress may promote epithelial cell loss through apoptosis, ferroptosis, or pyroptosis. Experimental studies using human intestinal models have implicated lipid peroxidation and ferroptosis in NPs-associated injury, with pharmacological inhibition of ferroptosis partially restoring cell viability (Sakib et al., 2026). Interestingly, the same study identified activation of Wnt/β-catenin signaling, which appeared to exert a compensatory protective effect rather than directly aggravating injury. These findings suggest that epithelial responses to MNPs involve both injury-associated and adaptive mechanisms.

Overall, current experimental evidence supports a framework in which MNPs-associated membrane perturbation and organellar stress contribute to metabolic dysfunction, impaired epithelial renewal, and regulated cell death. These processes may ultimately weaken epithelial integrity.

Organisms under disease conditions are suggested to exhibit increased susceptibility to environmental pollutants. Busch et al. (Busch et al., 2021) utilized an in vitro triple co-culture model (Caco-2/HT29-MTX-E12/THP-1) to simulate healthy and inflamed human intestinal epithelium. They observed no acute impact of MNPs on the endpoints of the healthy intestinal model; however, in the inflamed model, increased IL-1β release and epithelial cell loss were detected. Moreover, in vivo studies demonstrated that PS MNPs did not significantly affect mortality in healthy zebrafish and mice. In contrast, zebrafish and mice with IBD exhibited increased susceptibility to PS MNPs, as evidenced by mucus depletion, microvilli damage, and mitochondrial abnormalities in intestinal epithelial cells (Li et al., 2025b). Zheng et al. (Zheng et al., 2021) reported that MPs administration exacerbated acute DSS colitis and increased intestinal epithelial permeability. Collectively, these findings suggest that pre-existing intestinal inflammation may increase susceptibility to MNPs-induced intestinal injury. Accordingly, FMT recipients with underlying intestinal inflammatory conditions may be more vulnerable to the adverse effects of MNPs exposure, although this possibility remains to be directly investigated.

#### 4.3.3 Potential effects of MNPs on the immunological barrier

Mucosal immune responses constitute the host’s last defense line before systemic infection occurs due to translocation of commensal microbes across the intestinal barrier. Excessive cytokine production has been associated with poor FMT outcomes (Khoruts and Sadowsky, 2016). MNPs can promote inflammatory factor release by activating dendritic cells, macrophages, and other immune cells, thereby inducing or exacerbating local inflammation (Bazina et al., 2026; Giustarini et al., 2025; Hirt and Body-Malapel, 2020). It is hypothesised that MNPs-associated immune dysregulation may disrupt the intestinal microenvironment, potentially affecting donor microbiota engraftment and subsequent therapeutic FMT outcomes. Zhang et al. (Zhang et al., 2023c) showed that PS NPs significantly decreased key components of intestinal immunity, such as mucosal secretory IgA (sIgA), while inhibiting CD4^+^ and CD8^+^ T cell differentiation in mesenteric lymph nodes after 28 days of continuous exposure. Harusato et al. (Harusato et al., 2023) investigated environmentally relevant, non-spherical PET MNPs, administering mice with oral gavage of 3×10^4^ particles every three days for eight weeks (particle size 0.5-3.5 μm). Although no colonic epithelial damage, mucus barrier disruption, or elevation of proinflammatory cytokines (TNF-α, IL-6, IL-1β) were observed, transcriptomic analysis of intestinal CD45^+^ immune cells revealed significant metabolic reprogramming. Notably, oxidative phosphorylation and ROS pathways were enriched, while several immunoregulatory genes (e.g., Hbp1, Ccnd1, Cd247) were downregulated, suggesting that PET MNPs may perturb intestinal immune homeostasis without overt tissue inflammation (Harusato et al., 2023). Single-cell RNA sequencing of mouse intestines exposed to self-made NPs (500 nm) and MPs (2 μm) revealed that NPs more effectively activated intestinal macrophages. NPs induced lysosomal damage in intestinal macrophages, leading to IL-1 production and systemic immune responses (Yang et al., 2023). Li et al. (Li et al., 2025b) found that, aside from significant activation of Toll-like receptor signaling pathways in both PS NPs and PS MPs groups, NOD-like receptor and C-type lectin receptor signaling pathways were significantly enriched in the PS MPs group of IBD-model zebrafish, possibly reflecting enhanced apoptosis-associated immune activation that may aggravate inflammatory injury.

MNPs exposure may activate intracellular inflammatory signaling pathways that contribute to intestinal epithelial injury (Fig. 4). Mechanistically, the NF-κB pathway serves as a central node integrating upstream pattern recognition receptor activation with downstream inflammatory effector responses. Experimental evidence suggests that MNPs can activate NF-κB through several upstream mechanisms, including TLR4-associated signaling, focal adhesion kinase (FAK) activation, and ROS-dependent MAPK signaling. These mechanisms may contribute to epithelial barrier dysfunction through inflammatory mediator production and downstream regulation of tight-junction integrity.

**Fig. 4:**
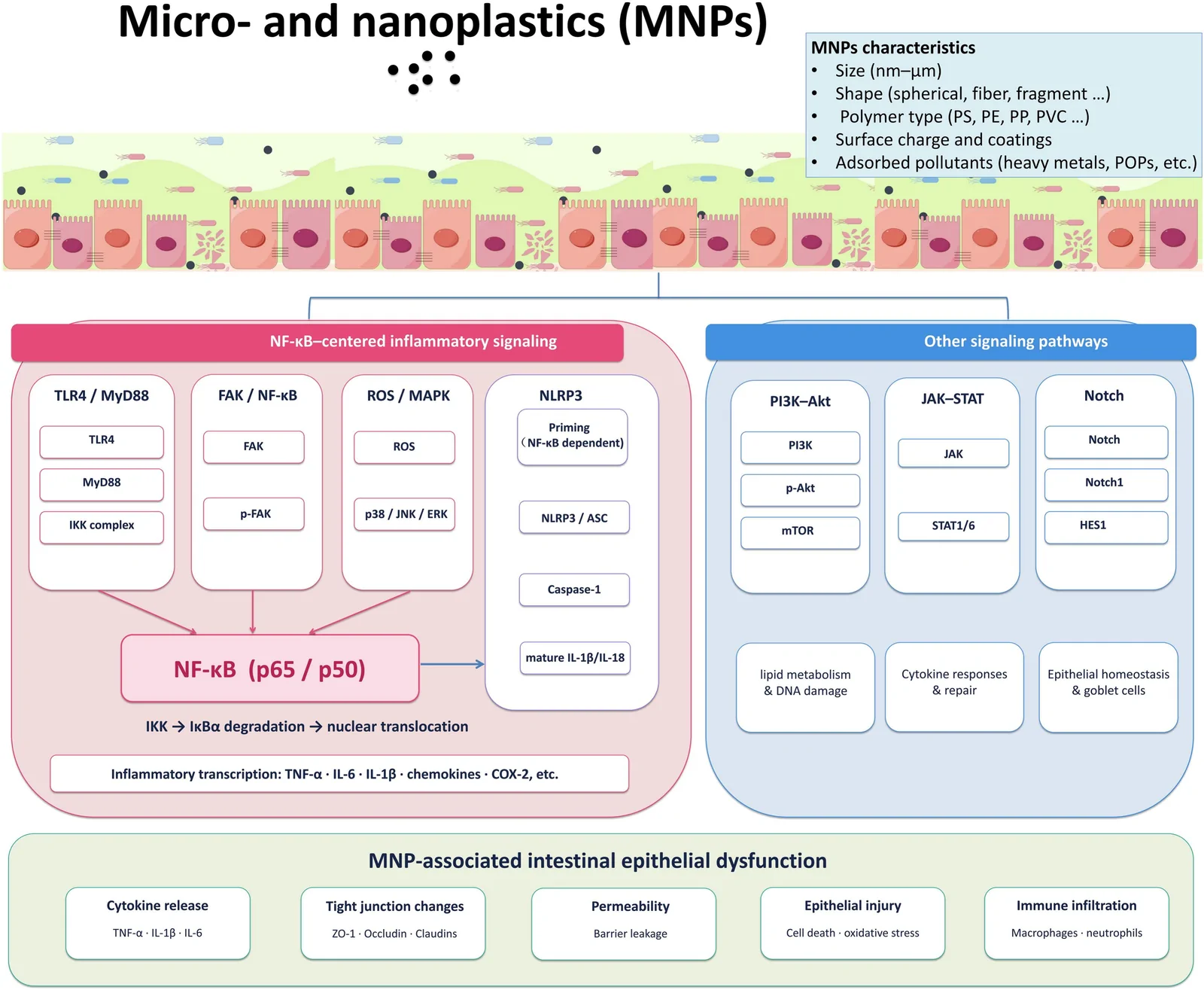
Molecular mechanisms underlying micro- and nanoplastics (MNPs)-associated intestinal epithelial inflammation and barrier dysfunction.

One of the best-characterized pathways involves TLR4/MyD88/NF-κB signaling. Feng et al. (Feng et al., 2025b) demonstrated that prolonged exposure to PS MPs induced colonic inflammation in rats, accompanied by increased TLR4/MyD88 expression, activation of NF-κB/COX-2-associated signaling, and elevated IL-1β, IL-6, and TNF-α levels. Inhibition of the investigated pathway attenuated the PS-MPs-associated increases in IL-6 and TNF-α, supporting its involvement in inflammatory activation. Li et al. (Li et al., 2026) reported that PS MPs fibers and beads activated TLR4/NF-κB/iNOS-associated signaling, accompanied by reduced expression of epithelial barrier-associated proteins, including ZO-1, occludin, E-cadherin, and MUC2. Their intervention experiments further supported a functional contribution of TLR4/NF-κB signaling to epithelial barrier injury. Notably, fiber-shaped particles additionally activated FAK/NF-κB/iNOS-associated signaling, suggesting that particle morphology may influence the upstream mechanisms of inflammatory activation.

Oxidative stress provides another mechanistic link between MNPs exposure and inflammatory activation. Zeng et al. (Zeng et al., 2024) demonstrated that PS MPs induced colonic oxidative stress and intestinal barrier dysfunction in mice, accompanied by increased inflammatory cytokine expression, elevated intestinal permeability, and reduced expression of tight junction proteins. Mechanistically, exposure to 5 μm PS MPs (100 μg/mL) increased ROS generation and NF-κB p65 phosphorylation in Caco-2 intestinal epithelial cells, accompanied by activation of NLRP3 inflammasome-related signaling and upregulation of IL-1β and MLCK. Following NF-κB-associated priming, the NLRP3 inflammasome can promote caspase-1-dependent maturation of IL-1β, thereby amplifying inflammatory responses. Additionally, Kim et al. (Kim et al., 2024) reported that PS NPs enhanced JAK/STAT-related transcriptional signatures and increased STAT1 and STAT6 phosphorylation in mouse intestines, accompanied by reduced tight junction protein expression, increased intestinal permeability, and exacerbated DSS-induced colitis. In addition, Tan et al. (Tan et al., 2026) identified an IFN-γ/STAT1/CXCL11-associated signaling response linked to CD3ε^+^ T-cell recruitment into the intestinal mucosa in zebrafish exposed to PS MPs. These findings suggest that MNPs-associated intestinal inflammation may involve both innate and adaptive immune responses. However, evidence from distinct experimental systems should not be interpreted as establishing a single, universally applicable signaling cascade.

In the context of FMT, these inflammatory and barrier-disrupting effects may be relevant because the recipient intestinal environment provides the host context in which donor-derived microorganisms must establish and maintain themselves. MNPs-induced intestinal barrier alterations, a plausible mechanism through which exposure might influence microbial engraftment or colonization persistence. Nevertheless, the studies discussed above primarily demonstrate MNPs-associated inflammatory and epithelial effects rather than direct impairment of FMT outcomes. Whether these changes reduce donor-derived microbial engraftment, compromise the durability of colonization, or diminish therapeutic efficacy requires direct evaluation in FMT models that jointly assess microbial persistence, recipient inflammation, and intestinal barrier function.

## 5 Conclusions and perspectives

The proposed donor “microbial seed” - recipient “intestinal soil” framework provides a conceptual basis for examining whether MNPs could influence biological processes relevant to FMT. Existing studies have demonstrated that MNPs can induce microbial dysbiosis, including enrichment of potentially harmful taxa and depletion of beneficial microorganisms, reduce the production of SCFAs and SBAs, impair mucus and tight junction integrity, and modulate inflammatory responses. These alterations may create an unfavorable intestinal environment that potentially influences donor microbiota engraftment and barrier recovery following FMT. However, these findings primarily establish MNPs-associated biological effects rather than impaired therapeutic FMT efficacy. Moreover, changes in individual microbial taxa should not be uniformly interpreted as beneficial or pathogenic, because their functional implications depend on microbial strain, community context, host characteristics, and disease state. Although MNPs-associated disturbances could theoretically modify the recipient intestinal environment or donor microbial characteristics, no direct evidence currently demonstrates that MNPs exposure reduces donor-strain engraftment, impairs microbiota-mediated functional recovery, or worsens clinical outcomes following therapeutic FMT. Accordingly, the proposed MNPs-FMT relationship should be regarded as a testable hypothesis that warrants direct experimental investigation.

The foremost research priority is to determine whether MNPs exposure actually influences FMT outcomes. To address this question, future investigations should move beyond studies of MNPs-induced toxicity and directly evaluate the relationship between MNPs exposure and therapeutic microbiota transplantation. Controlled animal experiments incorporating MNPs exposure and FMT could provide initial causal evidence by comparing donor microbial engraftment, intestinal microbial community reconstruction, metabolic functional recovery, barrier restoration, and disease-specific therapeutic outcomes across exposure groups. Importantly, donor and recipient exposure should be investigated separately to distinguish potential effects on the transplanted microbial community from those on the recipient intestinal environment. In parallel, prospective human studies should assess MNPs burdens in donors and recipients and examine their associations with longitudinal donor-strain engraftment, microbial functional recovery, and clinically defined FMT responses. Strain-resolved metagenomics, metabolomics, and repeated sampling before and after transplantation may help characterize the dynamics of microbial establishment and functional recovery. Such studies are essential to determine whether MNPs-associated biological alterations translate into measurable changes in FMT efficacy.

Prospective clinical studies incorporating quantitative assessments of MNPs exposure are needed to determine whether real-world MNPs burden is associated with the efficacy and safety of FMT. Accurate assessment of MNPs exposure during FMT represents an important methodological challenge. Although medical devices and healthcare environments may represent potential sources of iatrogenic MNPs, direct quantitative evidence demonstrating clinically meaningful exposure related to FMT procedures is currently lacking. Establishing standardized protocols for MNPs detection and quantification in donor fecal samples, recipient intestinal and FMT procedure-associates materials will be essential. Commonly used techniques, including fourier-transform infrared spectroscopy, raman spectroscopy, and pyrolysis-gas chromatography-mass spectrometry, provide information on polymer identity, particle abundance, and polymer mass. However, their analytical capabilities differ, and reliable characterization of NPs remains particularly challenging. Furthermore, unlike relatively simple environmental matrices, biological specimens contain complex organic components that can interfere with particle isolation, identification, and quantification. The lack of standardized sampling, contamination-control, recovery-assessment, and reporting protocols limits comparisons across studies. Future research should therefore prioritize the development and validation of sensitive, reproducible, and matrix-specific analytical methods capable of characterizing MNPs in biological samples. In addition, the identification of validated exposure-related biomarkers or surrogate indicators may provide a complementary approach when direct particle quantification is technically limited.

Another critical issue is the environmental relevance of current MNPs exposure models. Many preclinical studies have employed acute, high-dose exposure paradigms using single, pristine polymer types, particularly commercially available monodispersed PS microspheres. Although these models have provided valuable mechanistic insights, they may not adequately represent real-world MNPs long-time low-dose exposure scenarios. Recent analyses have highlighted substantial discrepancies between laboratory-generated PS particles and environmentally derived MNPs, including differences in exposure dose, polymer composition, particle size distribution, morphology, surface chemistry, plasticisers, and physicochemical properties (Bridgeman et al., 2025; Gouin et al., 2024; Schröter and Ventura, 2022). On the other hand, the gut microbiota composition and the intestinal microenvironment in animal models are largely different from those in humans, which may also influence the biological effects of ingested MNPs. Moreover, in human studies, diet, lifestyle, medication use, and other environmental factors can substantially confound the relationship between MNPs exposure and gut microbiota alterations, complicating the identification of MNPs-specific effects. Future preclinical studies should prioritize environmentally relevant exposure conditions, including chronic, low-dose exposure to aged and mixed-polymer MNPs, to better reflect real-world exposure scenarios. These approaches should be complemented by well-designed human studies that account for major confounding factors and, where feasible, prospective FMT studies incorporating MNPs exposure assessment to determine whether MNPs exposure is associated with donor microbiota characteristics, microbial engraftment, and therapeutic outcomes.

Potential MNPs exposure associated with FMT procedures represents a separate question that requires direct investigation. Although particle release from medical materials and contamination in healthcare environments have been reported, these observations do not demonstrate that clinically meaningful MNPs exposure occurs during FMT preparation or administration. Future studies could examine donor fecal material, processing equipment, intermediate preparations, and final transplant products using contamination-controlled analytical procedures to determine whether procedure-associated MNPs transfer occurs.

In conclusion, MNPs contamination represents a potential but currently unverified factor that may influence the safety and efficacy of FMT. Existing evidence supports the biological plausibility of interactions between MNPs exposure, gut microbial communities, intestinal barrier function, and mucosal immune regulation. However, whether these effects influence therapeutic FMT remains unknown. Future investigations should prioritize direct evaluation of FMT outcomes, supported by reliable quantification of MNPs exposure and environmentally relevant experimental models. Integrating quantitative exposure assessment with longitudinal microbial engraftment, functional recovery, and clinical outcomes will be essential to determine whether MNPs represent a meaningful modifier of FMT efficacy. Addressing this emerging concern requires interdisciplinary collaboration among environmental scientists, microbiologists, materials scientists, and clinicians. Such efforts will be essential for developing evidence-based strategies to optimize microbiota transplantation and ensure its safety and effectiveness.

## CRediT authorship contribution statement

**Qiuyue Tu:** Writing – review & editing, Writing – original draft, Visualization, Software, Resources, Methodology, Investigation, Formal analysis, Data curation, Conceptualization. **Huizhen Li:** Writing – review & editing, Visualization, Methodology, Investigation, Data curation, Conceptualization. **Jiaxin Li:** Writing – review & editing, Visualization, Methodology, Investigation, Data curation. **Zelin Feng:** Writing – review & editing, Visualization, Methodology, Investigation. **Xinyue Wei:** Writing – review & editing, Visualization, Methodology, Investigation. **Hetong Zhang:** Writing – review & editing, Validation, Investigation. **Qinghua Yi:** Writing – review & editing, Validation, Investigation. **Haojia Lin:** Writing – review & editing, Validation, Investigation. **Yanru Zhang:** Writing – review & editing, Visualization, Validation. **Yueting Li:** Writing – review & editing, Visualization. **Xiaoqin Li:** Writing – review & editing, Visualization. **Yu Wang:** Writing – review & editing, Visualization. **Ye Chen:** Writing – review & editing, Supervision, Funding acquisition, Conceptualization. **Qi Su:** Writing – review & editing, Supervision, Conceptualization. **Lei Wang:** Writing – review & editing, Supervision, Conceptualization. **Xiaocang Cao:** Writing – review & editing, Supervision, Funding acquisition, Conceptualization.

## Funding statement

This work was supported by the Shenzhen Medical Research Fund (number C2501011); 10.13039/501100001809National Natural Science Foundation of China (number 82270565); Shenzhen Key Laboratory of Gastrointestinal Microbiota and Disease, Shenzhen Science and Technology Program (number ZDSYS20220606100800002); National Key R&D Program of China (number 2024YFA1307102); and Sanming Project of Medicine in Shenzhen (number SZSM202411029).

## Declaration of competing interest

The authors declare that they have no known competing financial interests or personal relationships that could have appeared to influence the work reported in this paper.

## Data Availability

All information presented is obtained from previously published literature, which is fully cited in the manuscript.

## Appendix A Supplementary data

List of chemical compounds relevant to the review.

Demonstrated effects of micro- and nanoplastics and their hypothesized implications for fecal microbiota transplantation.
