# Supplementary material for: Micro- and nanoplastics and fecal microbiota transplantation: a conceptual framework linking gut dysbiosis and intestinal barrier dysfunction
Source: Int J Pharm X. 2026 Nov 3;12:100699. doi: 10.1016/j.ijpx.2026.100699 (PMC13769601; doi:10.1016/j.ijpx.2026.100699)
Supplement: Supplementary file 1 [file mmc1.docx]

chemical compounds studied in the article

Microplastics

Nanoplastics

Polystyrene

polyvinyl chloride

Polyethylene

Polyethylene terephthalate
