# Supplementary material for: Micro- and nanoplastics and fecal microbiota transplantation: a conceptual framework linking gut dysbiosis and intestinal barrier dysfunction
Source: Int J Pharm X. 2026 Nov 3;12:100699. doi: 10.1016/j.ijpx.2026.100699 (PMC13769601; doi:10.1016/j.ijpx.2026.100699)
Supplement: Supplementary file 2 [file mmc2.docx]

Supplementary material

Table S1 Demonstrated effects and hypothesized effects of MNPs^1^ on FMT

| MNP-induced alterations | Demonstrated effects | Hypothesized effects |
| --- | --- | --- |
| **Altered microbial diversity and community structure** | • MNP exposure alters α- and β-diversity and microbial community composition in multiple animal models.[1]  • Human evidence remains limited and heterogeneous.[2] | • Reduced microbial diversity may create an unfavorable ecological niche for donor microbiota colonization and persistence. |
| **Gut bacteria** | • Animal studies consistently show altered gut bacterial composition after MNPs exposure, including reduced beneficial taxa (e.g., Allobaculum) and increased opportunistic/inflammation-associated taxa.[3]  • Limited human evidence suggests higher fecal MNPs burden is associated with increased Veillonella and Alistipes and decreased Coprococcus.[4] | • Loss of beneficial bacteria and enrichment of potentially harmful taxa may reduce donor microbiota stability and impair microbial engraftment.  • MNPs-induced dysbiosis may decrease the resilience of the recipient gut ecosystem. |
| **Gut fungal** | • Mouse studies demonstrate that PS MNPs alter fungal community composition, including changes in Scedosporium, Pichia, Candida, Curvularia, Malassezia, and Cladosporium.[5]  • Preliminary human data suggest possible changes in fungal richness and potentially pathogenic fungal taxa.[4] | • MNP-induced fungal imbalance may disrupt mucosal immune homeostasis and influence bacterial–fungal interactions. |
| **Gut phages** | • Limited evidence from zebrafish models suggests PS MNPs exposure alters specific phage populations (e.g., increased Listeria phage WIL-3).[6]  • Human evidence is currently unavailable. | • MNPs-associated phages dysbiosis may influence bacterial community stability, donor microbiota engraftment. |

Table S1 Continue

| MNP-induced alterations | Demonstrated effects | Hypothesized effects |
| --- | --- | --- |
| **Reduced SCFAs^2^ production** | • Multiple animal studies show decreased SCFAs production following MNPs exposure.[7, 8]  • Human evidence is inconsistent and insufficient.[9] | • Reduced SCFAs availability may impair epithelial repair, immune regulation, and colonization of beneficial donor bacteria. |
| **Altered bile acid metabolism** | • Animal studies demonstrate MNPs-induced disruption of bile acid metabolism, including reduced secondary bile acids and increased primary/secondary bile acid imbalance.[10] | • Reduced secondary bile acid production may negatively affect colonization resistance. |
| **Gut barrier and immune consequences mediated by microbiota** | • Animal studies demonstrate that MNPs exposure can impair mucus production, tight junction integrity, and promote inflammation.[11, 12] | • A compromised recipient intestinal barrier (“soil”) may reduce donor microbiota colonization and diminish FMT effectiveness. |

^1^MNPs: micro- and nanoplastics

^2^SCFAs: Short-chain fatty acids

1.	Chen K, Wang L, Liu J et al. The ant that may well destroy a whole dam: a systematic review of the health implication of nanoplastics/microplastics through gut microbiota. Crit Rev Food Sci Nutr 2025; 65: 6941-6962. doi:10.1080/10408398.2025.2453632

2.	Yang XY, Zhang ZW, Chen GD et al. Gut microbiome remodeling induced by microplastic exposure in humans. Gut Microbes 2026; 18: 2617696. doi:10.1080/19490976.2026.2617696

3.	Zhang Z, Chen W, Chan H et al. Polystyrene microplastics induce size-dependent multi-organ damage in mice: Insights into gut microbiota and fecal metabolites. Journal of Hazardous Materials 2024; 461. doi:10.1016/j.jhazmat.2023.132503

4.	Hong Y, Feng Y, Yan T et al. Take-out food enhances the risk of MPs ingestion and obesity, altering the gut microbiome in young adults. Journal of Hazardous Materials 2024; 476. doi:10.1016/j.jhazmat.2024.135125

5.	Gao B, Shi X, Li S et al. Size-dependent effects of polystyrene microplastics on gut metagenome and antibiotic resistance in C57BL/6 mice. Ecotoxicology and Environmental Safety 2023; 254. doi:10.1016/j.ecoenv.2023.114737

6.	Teng M, Li Y, Zhao X et al. Vitamin D modulation of brain-gut-virome disorder caused by polystyrene nanoplastics exposure in zebrafish (Danio rerio). Microbiome 2023; 11. doi:10.1186/s40168-023-01680-1

7.	174 Zhang Z, Xu M, Wang L et al. Continuous oral exposure to micro- and nanoplastics induced gut microbiota dysbiosis, intestinal barrier and immune dysfunction in adult mice. Environment International 2023; 182. doi:10.1016/j.envint.2023.108353

8.	Kang H, Huang D, Zhang W et al. Propionic acid/FBP1 is involved in polystyrene nanoplastic-induced cardiac injury via the gut-heart axis. Part Fibre Toxicol 2025; 22: 10. doi:10.1186/s12989-025-00626-9

9.	Tilves C, Zhao HJ, Differding MK et al. Associations of Plastic Bottle Exposure with Infant Growth, Fecal Microbiota, and Short-Chain Fatty Acids. Microorganisms 2023; 11. doi:10.3390/microorganisms11122924

10.	168 Wen J, Sun H, Yang B et al. Environmentally Relevant Concentrations of Microplastic Exposure Cause Cholestasis and Bile Acid Metabolism Dysregulation through a Gut-Liver Loop in Mice. Environmental Science & Technology 2024; 58: 1832-1841. doi:10.1021/acs.est.3c07108

11.	Zhang Z, Xu M, Wang L et al. Continuous oral exposure to micro- and nanoplastics induced gut microbiota dysbiosis, intestinal barrier and immune dysfunction in adult mice. Environ Int 2023; 182: 108353. doi:10.1016/j.envint.2023.108353

12.	215 Zeng G, Li J, Wang Y et al. Polystyrene microplastic-induced oxidative stress triggers intestinal barrier dysfunction via the NF-κB/NLRP3/IL-1β/MCLK pathway. Environmental pollution (Barking, Essex : 1987) 2024; 345: 123473. doi:10.1016/j.envpol.2024.123473
